# Antimicrobial resistance and COVID-19: Intersections and implications

**DOI:** 10.7554/eLife.64139

**Published:** 2021-02-16

**Authors:** Gwenan M Knight, Rebecca E Glover, C Finn McQuaid, Ioana D Olaru, Karin Gallandat, Quentin J Leclerc, Naomi M Fuller, Sam J Willcocks, Rumina Hasan, Esther van Kleef, Clare IR Chandler

**Affiliations:** 1 AMR Centre, London School of Hygiene and Tropical Medicine (LSHTM)LondonUnited Kingdom; 2 Centre for Mathematical Modelling of Infectious Diseases (CMMID), LSHTMLondonUnited Kingdom; 3 Department of Infectious Disease Epidemiology, Faculty of Epidemiology and Public Health, LSHTMLondonUnited Kingdom; 4 TB Centre, LSHTMLondonUnited Kingdom; 5 Department of Health Services Research and Policy, Faculty of Public Health and Policy, LSHTMLondonUnited Kingdom; 6 Clinical Research Department, Faculty of Infectious and Tropical Diseases, LSHTMLondonUnited Kingdom; 7 Biomedical Research and Training InstituteZambezi RiverZimbabwe; 8 Department of Disease Control, Faculty of Infectious and Tropical Diseases, LSHTMLondonUnited Kingdom; 9 Department of Infection Biology, Faculty of Infectious and Tropical Diseases, LSHTMLondonUnited Kingdom; 10 Department of Pathology and Laboratory Medicine, Aga Khan UniversityKarachiPakistan; 11 Department of Immunology and Infection, Faculty of Infectious and Tropical Diseases, LSHTMLondonUnited Kingdom; 12 Department of Public Heath, Institute of Tropical MedicineAntwerpBelgium; 13 Department of Global Health and Development, Faculty of Public Health and Policy, LSHTMLondonUnited Kingdom; University of PittsburghUnited States; Pennsylvania State UniversityUnited States

**Keywords:** COVID-19, antimicrobial resistance, global health

## Abstract

Before the coronavirus 2019 (COVID-19) pandemic began, antimicrobial resistance (AMR) was among the top priorities for global public health. Already a complex challenge, AMR now needs to be addressed in a changing healthcare landscape. Here, we analyse how changes due to COVID-19 in terms of antimicrobial usage, infection prevention, and health systems affect the emergence, transmission, and burden of AMR. Increased hand hygiene, decreased international travel, and decreased elective hospital procedures may reduce AMR pathogen selection and spread in the short term. However, the opposite effects may be seen if antibiotics are more widely used as standard healthcare pathways break down. Over 6 months into the COVID-19 pandemic, the dynamics of AMR remain uncertain. We call for the AMR community to keep a global perspective while designing finely tuned surveillance and research to continue to improve our preparedness and response to these intersecting public health challenges.

## Introduction

The coronavirus disease 2019 (COVID-19) outbreak, caused by the SARS-CoV-2 virus, was declared a pandemic by the World Health Organization (WHO) on 11 March 2020 (Ghebreyesus, 2020) and has reshaped the world. Antimicrobial resistance (AMR), a similarly cross-sectoral challenge which has received increased international attention since 2015 (WHO, 2015a), had already been named a priority for global public health for the year 2020 (World Health Organization, 2020). Based on previous estimates of the number of deaths from multidrug-resistant tuberculosis (MDR-TB) alone (182,000 in 2019; WHO, 2020d), thought to contribute a third of the burden (Global Fund, 2019), AMR is likely to have caused a third as many deaths as COVID-19 in 2020 (1.8 million; WHO, 2020h).

As the AMR research community learns more about the mechanisms of SARS-CoV-2 transmission, its interaction with other diseases, the policy responses to COVID-19 around the globe, and the behaviours associated with COVID-19 interventions, the direct and indirect impacts of COVID-19 on AMR and vice versa are becoming increasingly clear. Drawing from the expertise of the interdisciplinary AMR Centre at the London School of Hygiene and Tropical Medicine (amr.lshtm.ac.uk), we review the emerging evidence on this intersection. In doing so, we build on the literature exploring this intersection from single dimensions such as the impact on particular pathogens or systems. First, we examine the effect of AMR on the care of those with COVID-19. Second, we explore the impact of COVID-19 on the emergence, transmission, and burden of AMR through three dimensions: antimicrobial usage, infection prevention, and health system changes. Finally, we ask what this means for AMR research priorities and what the future holds for AMR in the ‘new normal’ of living with COVID-19.

## Impact of AMR on COVID-19 clinical care

Patients with COVID-19 may receive antimicrobial therapy for two main reasons. First, COVID-19 symptoms can resemble bacterial pneumonia. Diagnostics used to distinguish viral from bacterial pneumonia may prove ineffective or have turnaround times of hours or days when immediate treatment is needed. For example, faster tests, such as diagnostics measuring C-reactive protein – a biomarker that is elevated in bacterial infections but typically not in viral ones – may in fact be increased in patients with COVID-19 (Sproston and Ashworth, 2018). As a result, many patients hospitalised with COVID-19 will be prescribed empiric antibiotics, often in the absence of a microbiological confirmation of the diagnosis (Langford et al., 2021).

Second, patients with COVID-19 may acquire secondary co-infections which require antimicrobial treatment. Several evidence reviews suggest that the secondary bacterial infection rates are low (<20%) (Langford et al., 2020; Lansbury et al., 2020; Rawson et al., 2020b), but more, better data are needed to provide a better understanding of the occurrence of co-infections and pathogens involved, alongside the impact of underlying patient risk factors. In many of these studies, secondary infections were subsidiary endpoints and hence, moving forward, standardised definitions and diagnostic criteria should be used to perform more in-depth analysis of microbiological, resistance and antimicrobial usage data, where diagnostic laboratory infrastructure exists.

Local stewardship guidance, often based on local antimicrobial susceptibility data where available, influences a clinician’s choice of antimicrobial for their patients. Empiric treatment intends to cover a wide range of suspected organisms. Hence, AMR will influence the choice of antimicrobials prescribed to those with COVID-19. Clinicians are therefore challenged with competing priorities: prescribing a broad enough spectrum antimicrobial to ensure the organism is sensitive, while at the same time avoiding the unnecessary use of antimicrobials, particularly those of last resort, when a more commonly used or narrower-spectrum antimicrobial would suffice. Inappropriate treatment in either direction has been associated with increased risk of mortality (Gutiérrez-Gutiérrez et al., 2017; Paul et al., 2010).

Concern about potential infections with resistant pathogens could lead to unnecessary empiric prescribing of last resort antimicrobials to patients with COVID-19. For example, in areas where carbapenem resistance is high, antibiotics with less favourable safety profiles such as colistin may be recommended as a first-line treatment for suspected Gram-negative infections (Torres et al., 2017). This may result in more frequent adverse events and worse clinical outcomes in patients with COVID-19. Conversely, if recommendations for empiric treatment are not tailored to the local AMR prevalence, patients with co-infections may receive ineffective treatment which may in turn result in increased mortality and healthcare costs.

## COVID-19 impact on AMR

The evolution of AMR in a population is determined by three components: emergence-, transmission-, and population-level infection burden.

AMR *emergence* can be driven by selective pressures on microbial populations within humans, animals, or in the environment. Subject to the ‘drug and bug’ of concern, such selective pressures facilitate resistance acquisition mechanisms such as point mutation or horizontal transfer of genes encoding resistance to one or several antibiotics (Davies and Davies, 2010). Environmental conditions and behaviours may subsequently enable or prevent *transmission* of these emerged antimicrobial-resistant organisms (AROs) between humans, animals, and environments (Robinson et al., 2016). The *burden* of illnesses caused by AROs will then depend upon the number and nature of infections, and the availability, effectiveness, and safety of alternative treatments.

**Figure 1.**
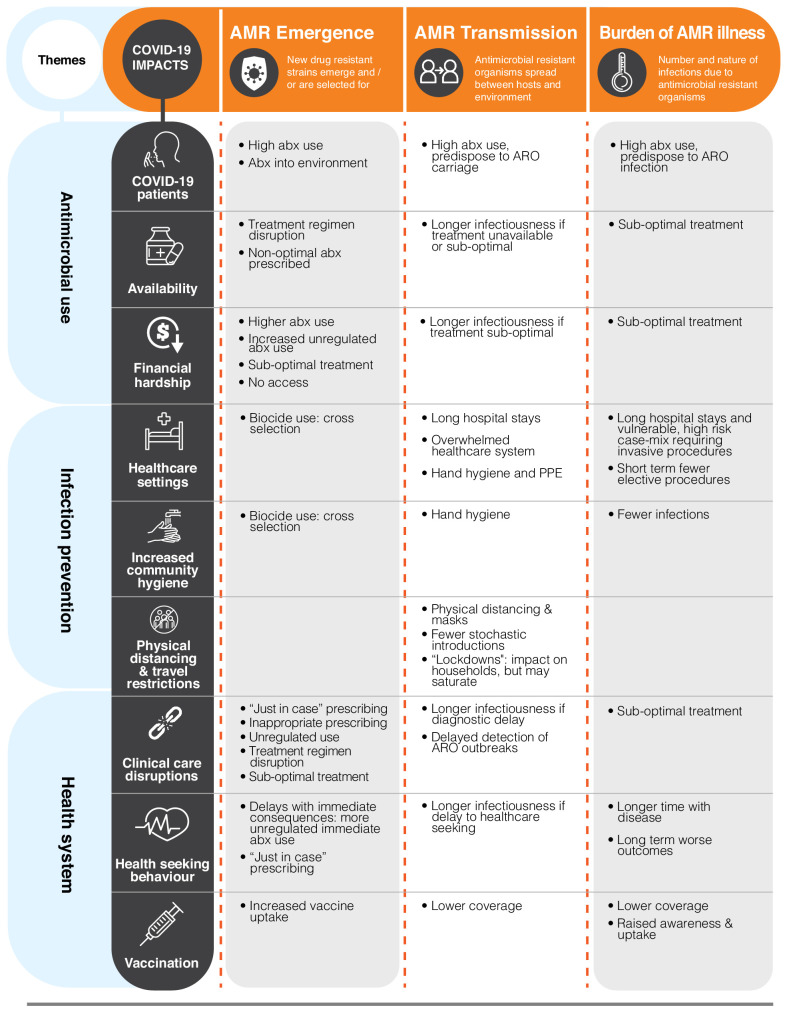
A grid of examples of the interactions between the dimensions of change brought on by the coronavirus disease 2019 (COVID-19) pandemic (rows) and the key components in the evolution of antimicrobial resistance (AMR, columns). This is not exhaustive but highlights the variability across the intersection and can be used to support the identification of research priorities. For more information, see the related dimension of change section in the text. ARO: antimicrobial-resistant organism; PPE: personal protective equipment.

COVID-19 has the potential to affect all three of these components through the direct or indirect consequences of pandemic responses. Government-led interventions undertaken to address COVID-19 have varied, but have included combinations of domestic and international travel restrictions; school, workplace, and non-essential service closures; physical distancing measures; and mask wearing (Blavatnik School of Government, 2020). Consequences of these changes include supply chain disruptions; healthcare access disruptions, financial impacts, and rising inequalities. Here, we group these interventions into three dimensions (antimicrobial use, infection prevention, and health system changes) in order to highlight the ways in which different dimensions of change in response to COVID-19 can affect the emergence, transmission, and burden of AMR (Figure 1).

### Antimicrobial use changes

Fundamentally, changes in antimicrobial use will drive changes in AMR. In this section, we outline the direct impact of COVID-19 on antimicrobial use in those with COVID-19 and then knock-on effects of COVID-19 in terms of antimicrobial availability and financial hardship.

#### Antimicrobial use in those with COVID-19

As many as 70% of patients with COVID-19 receive antimicrobials either in the outpatient or inpatient setting (ISARIC, 2020; Langford et al., 2021). This may favour the emergence of AMR.

There are several reasons patients with COVID-19 will receive antimicrobials (as outlined above). It is also likely that the pandemic may result in a large cohort of COVID-19 survivors with residual post-COVID-19 chronic lung disease (such as interstitial lung disease and bronchiectasis) who will be at risk of acute exacerbations (George et al., 2020). Long-term sequelae of individuals recovered from moderate to severe COVID-19 is currently unknown, but extensive lung damage has been reported on high-resolution chest computed -tomography several weeks into the illness (Zhao et al., 2020). In patients with post-COVID-19 structural lung damage, frequent courses of antibiotics and hospital admissions may lead to increased risk of colonisation and infection with resistant organisms such as *Pseudomonas aeruginosa* (Rodrigo-Troyano and Sibila, 2017).

Antimicrobials have been used for their presumed direct effect on SARS-CoV-2. This may lead to resistance emergence in co-infecting or co-colonising pathogens (‘bystander selection’) (Tedijanto et al., 2018). For example, despite mounting evidence of a lack of efficacy (Kashour et al., 2021; UKRI, 2020; WHO, 2020e), the antimalarial drug chloroquine has been used to treat COVID-19. This is of particular concern in high non-*Plasmodium falciparum* prevalence malaria settings, where chloroquine remains the drug of choice for malaria (WHO, 2015b) and its use for COVID-19 may encourage chloroquine resistance emergence.

Because a patient with COVID-19 can present with non-specific symptoms (e.g. fever and/or persistent cough), these could be mistaken for other disease such as malaria (Chanda-Kapata et al., 2020) or tuberculosis (TB) (The Lancet Hiv, 2020), and vice versa. These overlapping symptoms may result in inappropriate prescribing or a lack of prescribing and misdiagnosis, depending on COVID-19 prevalence. This could impact future drug resistance levels of other pathogens, as mentioned above. The importance of such bystander selection has already been seen following the widespread use of azithromycin for WHO-recommended mass drug administration campaigns for trachoma (O'Brien et al., 2019). This is particularly relevant for COVID-19 as azithromycin has been proposed as a potential therapy.

As the evidence mounts on the lack of efficacy of hydroxychloroquine, chloroquine, and azithromycin for treatment of COVID-19, their initial use has now been halted in some settings (e.g. in the US [Acosta et al., 2020; Bull-Otterson et al., 2020; Kadri et al., 2020]). However, misinformation (Arshad et al., 2020), the lack of other treatment, combined with the severity of the illness, and a desire to try all avenues have led to several settings continuing to use these drugs. For example, in many African countries, despite WHO recommendations, hydroxychloroquine, chloroquine, and azithromycin were still being advised for use in the summer of 2020 and may be still being used off-label (Abena et al., 2020; Belayneh, 2020). In India, it appears to still be guidance to use hydroxychloroquine as a prophylactic for ‘healthcare workers’ (National Taskforce for COVID-19, India, 2020).

In community settings, cheap, rapid tests may detect SARS-CoV-2 infections earlier (Mina et al., 2020), especially in the face of unspecific symptoms, and hence prevent some antibiotic use. This will be particularly important in low- and middle-income settings (WHO, 2020m) where antibiotics can be obtained without prescription and where testing is a priority while vaccines may take longer to be more broadly used. However, it remains to be seen whether the affordability, availability, and acceptability of these tests and the often stigmatised diagnosis will lead to sufficient uptake that they remove a substantial proportion of such empiric antibiotic use. Moreover, some current diagnostics for asymptomatic testing in the community, such as lateral flow tests, remain insensitive and risk providing ‘false negatives’ with a sense of security that is not accurate (Mahase, 2020).

If prolonged, changes in antimicrobial use in humans during the COVID-19 crisis could mean increased antimicrobial production, which may accelerate changes in antimicrobial concentrations in the environment and, consequently, affect selective pressures for AMR in water and soil systems (WHO, 2020a). This in turn could lead to selection for AMR within animals and potentially increase ARO prevalence within the animal-based food supply chain, although the impact will vary by antimicrobial and pathogen. Due to the One Health nature of AMR, this impact on AMR in animals and the environment may feedback to levels of AMR infections in humans, although the extent to which this could happen remains uncertain (Robinson et al., 2016; Tang et al., 2017).

Presumptive antibiotic use is also happening more due in part to fractured health systems – this is covered in a later section where we focus on the impact of disrupted clinical care.

#### Antimicrobial availability

COVID-19 has affected the availability of antimicrobials by disrupting both supply chains and global manufacturing of antimicrobials, leading to knock-on changes in usage patterns.

The fragility of the antibiotic pipeline was highlighted recently when disruption within a single Chinese factory resulted in an international shortage of piperacillin-tazobactam (Davis, 2017). Concern about shortages has led the European Medicines Agency to lead ‘urgent and coordinated action to prevent and mitigate drug shortages within the EU’ (EMA, 2020). In some countries, such as the UK, 80–90% of generic medicines are imported (Business and Energy and Industrial Strategy Committee, 2018), making healthcare systems susceptible to travel restrictions or delayed supply chain issues. Indeed, most countries are net importers of antibiotics (Simoes and Hidalgo, 2011). Countries that manufacture and export antibiotics are facing increased home market demands due to COVID-19 which may lead to a reduction in exports, as seen in India (Government of India, 2020; Guerin et al., 2020). This could lead to a call for more antibiotic manufacturing outside of the Indian and Chinese manufacturing hotspots. As COVID-19 is also likely to lead to changes in the use of antibiotics, this will have a similar effect on supply chain shortages; 10 manufacturers have reported shortages of azithromycin to the US Food and Drug Administration, potentially because of its use for COVID-19 treatment (US Food and Drug Administration, 2020).

Low- and middle-income country (LMIC) settings are particularly vulnerable to supply chain issues where weak health systems and poor quality control of medicines are barriers to antimicrobial access (Frost et al., 2019; Laxminarayan et al., 2016; Pokharel et al., 2019). Uneven supplies of different antimicrobials across all income settings (Pauwels et al., 2015; Tickell et al., 2019) raise concerns about AMR emergence due to suboptimal antibiotic use (Access to Medicine Foundation, 2018). Concerns also arise over the sources of antimicrobials that become available in crisis scenarios, and the potential that fragmented systems combined with reduced regulatory control increase the risk that substandard or counterfeit drugs may become more commonly used. Such medicines may not only be the inappropriate choice for the illness but may have suboptimal concentrations of antimicrobial, favouring resistance, and can potentially be toxic to the patients (Kelesidis and Falagas, 2015; Pisani, 2015).

More widely, an increased use of broad-spectrum antibiotics, or inappropriate antimicrobials as alternatives when the recommended or usual antimicrobial is unavailable, will need to be monitored and responded to. The potential consequences are more resistance emerging to reserved, second-line antibiotics (e.g. higher tiers of the AWaRe classification; WHO, 2019) or increased resistance to broad-spectrum antibiotics. In the case of TB, which requires prolonged multidrug therapy, one consequence of drug shortages will be short-term treatment interruptions potentially leading to increased risk of treatment failure and development of resistance (Adepoju, 2020). Thus the AMR picture is likely to be different in the future: resistance to some hard to access antimicrobials may decrease, while resistance to others may increase.

#### Financial hardship

Fiscal and monetary measures introduced to manage different aspects of the pandemic may serve to either raise or lower the risk of AMR – depending on which measures are taken, for how long, and how strong the support on offer is.

The wider societal impact of the COVID-19 pandemic may be a lowering of the overall wealth of nations; another ‘financial crash’ (Sumner et al., 2020; World Bank, 2020). In high-income country (HIC) settings, where prescriptions for antibiotics are required, it is likely that a reduction in income will be associated with poorer health; such reductions have previously been associated with higher rates of both respiratory infections and antibiotic prescribing in Scotland (Covvey et al., 2014), Sweden (Nilsson and Laurell, 2005), and, more recently, in the US (Kissler et al., 2020). Similarly, less prosperous socioeconomic areas within such countries have experienced a higher burden of COVID-19 (Ahmed et al., 2020; Office for National Statistics, 2020). This could lead to an amplification effect, with disproportionate increases in AMR prevalence in those places hardest hit by COVID-19.

In LMIC settings, where there is already less regulation of antimicrobial purchasing, the financial shocks due to COVID-19 policies may increase the need for people to use over-the-counter services including for unregulated antimicrobials in an attempt to reduce healthcare expenditure (Frost et al., 2019). This presents a higher risk of subtherapeutic doses of drugs and shortened courses of antimicrobials, as well as increased mortality (Laxminarayan et al., 2013). Either option could select for resistance. On the other hand, the economic reverberations of this crisis mean that health systems that already struggle to provide sufficient access to antimicrobials (Frost et al., 2019; Laxminarayan et al., 2016) may face further challenges to deliver, and for individuals pushed into greater poverty, access to medicines at all may become even less affordable (Mendelson et al., 2016; Nayiga et al., 2020). This may result in a potential increase in mortality, while such access has been shown to correlate with higher burden of preventable infections such as community-acquired pneumonia in children under 5 (Laxminarayan et al., 2016). Thus, a combination of suboptimal antibiotic use with declining access may enhance selection pressure in favour of AMR, while at the same time increasing mortality from infections that could be averted with antibiotics.

The impact of COVID-19 and the policies intended to mitigate its spread are also creating and exacerbating health inequities (Mendenhall, 2020) across all the Cochrane PROGRESS Equity groups (Cochrane, 2020). For example, gender inequalities impact both COVID-19 and AMR due to the uneven distribution of infectious disease prevalence and burden (Horton et al., 2016; McQuaid et al., 2020; Schröder et al., 2016; Wenham et al., 2020; Zanichelli et al., 2019).

The pandemic has also highlighted racial inequality; in particular, the Black, Asian, and minority ethnic (BAME) categorisation has been used in some countries (Hu, 2020) to show how these populations suffer from both a greater economic impact and worse health outcomes from COVID-19 (Public Health England, 2020a). This twofold risk is amplified by lower socioeconomic status (Ahmed et al., 2020), with, for example, challenges in physical distancing. Emerging evidence suggests that in the UK BAME populations also have higher rates of circulating AMR in the community (Lishman et al., 2016). The posited mechanisms of this association are frequent travel to areas with higher endemic AMR prevalence such as the Indian subcontinent, differences in awareness of appropriate antibiotic use, and even differences in diet, but research is ongoing in this burgeoning field (Lishman et al., 2016). These inequalities combine with a lack of access to appropriate health care due to the COVID-19 crisis to suggest we may see subpopulations with dual severe issues of COVID-19 and AMR (syndemics).

### Infection prevention

Many of the interventions to prevent the spread of SARS-CoV-2 should also limit transmission of ARO. It has been postulated that countries with the greatest AMR burden are those in which spread of pathogens, rather than overuse of antimicrobials, is the dominant factor (Collignon et al., 2018). Hence, with increased infection prevention control (IPC) due to COVID-19, AMR prevalence may substantially decrease (Collignon and Beggs, 2020).

For MDR-TB – the largest single disease component of the global AMR burden (The Review on Antimicrobial Resistance, 2016) – the majority of cases result from transmission events rather than acquisition of spontaneous resistance emerging during treatment (Kendall et al., 2015). However, as with many of the interactions outlined here, the picture is not so simple. For example, with increased physical distancing many opportunities for transmission may be removed, but transmission within a household may be amplified. Similarly, healthcare settings may see increases in healthcare-associated infections caused by ARO due to COVID-19 pressures, despite an initial decrease in elective procedures. Below we detail the potential interaction with COVID-19-related IPC measures for different settings of ARO spread.

#### Infection burden in healthcare settings

Patients with severe COVID-19 may be hospitalised for prolonged periods of time (Rees et al., 2020), and hence are at high risk for acquiring nosocomial infections (Asensio et al., 1996; Oztoprak et al., 2006; Wolkewitz et al., 2008). Moreover, patients with COVID-19 may require multiple courses of broad-spectrum antibiotics, mechanical ventilation, other organ support, and/or other invasive devices. This increases exposure to, and risk of, infections with hospital-associated pathogens that are often highly resistant such as methicillin-resistant *Staphylococcus aureus* (MRSA), *P. aeruginosa, Candida auris,* and *Acinetobacter baumannii* (Chowdhary et al., 2020; Perez et al., 2020; Punjabi et al., 2020). Early anecdotal evidence from two acute care facilities in New York and Missouri reported three- to fourfold increases in central line-associated bloodstream infection rates, likely related to a shift towards a higher risk case-mix (McMullen et al., 2020). The hardships and resource strains due to COVID-19 have also affected antimicrobial stewardship activities (Hsu, 2020; De Waele et al., 2021; Ginsburg and Klugman, 2020; Lynch et al., 2020; Seaton, 2020). It is known that antimicrobial stewardship programmes in hospitals have the potential to significantly reduce incidence of infections with ARO (Baur et al., 2017), with the main aim being to minimise the risk of antibiotic misuse leading to AMR. Thus, COVID-19 may directly increase the number of infections with ARO, with the specific problematic ARO being setting dependent (Monnet and Harbarth, 2020).

Other factors may also contribute to an increased risk of nosocomial infection: shortages of personal protective equipment (PPE), staff shortages due to self-isolation and increased demand, fatigue, and deployment of inexperienced staff with only basic training (Donà et al., 2020). More nosocomial transmission of ARO, or of SARS-CoV-2, may occur during periods of high COVID-19 incidence if hospitals are overwhelmed by patients with COVID-19 (as was seen in Italy) (Schnirring, 2020). Some of these factors likely contributed to an increased acquisition of MRSA during the SARS outbreak in 2003 in an intensive care unit in China (Yap et al., 2004). In general, COVID-19 has led to competition for side room utilisation and diversion of PPE (Rawson et al., 2020b) from those requiring isolation due to colonisation or infection with other pathogens.

Alternatively, hospital-acquired AROs may have seen a break in transmission due to fewer patients in secondary care or changing patient profiles such as fewer elective surgical interventions. Enhanced infection prevention, PPE usage and control measures, as well as targeted hygiene, in response to the COVID-19 pandemic (Monnet and Harbarth, 2020; WHO, 2020i; WHO, 2020l), will help prevent infections in general and thus will contribute to limiting the spread of AMR both through the prevention of ARO spread and the reduction in antimicrobial prescribing for infections (Donà et al., 2020). Despite some concerns that enhanced biocide use could lead to cross-resistance development (Getahun et al., 2020), it is not unlikely that the COVID-19 response will prevent infections: early signs of nosocomial infection decline in English mandatory reporting data have been reported, but will have to be considered with care following a decrease in reporting (Public Health England, 2020b).

#### Increased hygiene in the community

Inadequate hygiene or sanitation in home environments may favour the transmission of ARO (Maillard et al., 2020). Studies have shown that handwashing alone could prevent 30% of diarrhoeal episodes in LMICs (Ejemot-Nwadiaro et al., 2015). As a result, increased hand hygiene to prevent SARS-CoV-2 transmission could reduce diarrhoeal incidence and hence the high number of antibiotic courses given to children with diarrhoea in LMICs (up to 50% with diarrhoea receive antibiotics) (Fink et al., 2020). Transmission of zoonotic ARO is likely to be reduced by increased hygiene and food safety measures in settings like wet markets in China (Hernández et al., 2020; Nhung et al., 2018).

To improve hygiene, there is likely to be an increased use of biocides across community and healthcare settings. It has been shown that low-level use of these agents can select for resistance both to the biocides and to antimicrobials (Caselli, 2017; Hardy et al., 2018; Hora et al., 2020; Kampf, 2018; Maillard et al., 2020; McCarlie et al., 2020; Wales and Davies, 2015), though the mechanisms and importance of this in driving the spread of AMR are unclear.

#### Physical distancing measures and travel restrictions

‘Lockdowns’ and social or physical distancing measures, such as closing childcare centres, will reduce the transmission opportunities for many pathogens beyond SARS-CoV-2, including AROs. For example, numbers of invasive bacterial infections appear to have decreased in 2020 (Brueggemann et al., 2020), such as Invasive Group A Streptococcal Infections in the UK (Public Health England, 2020a). This could potentially be linked to reduced transmission, although reporting issues may also play a part and most of these data are from high-income European settings. Similarly, many settings have seen far reduced influenza infection levels (WHO, 2020n), likely to lead to linked decreases in inappropriate and appropriate antibiotic use. The impact of physical distancing, as well as increased awareness of infection prevention measures, on ARO, as well as ‘flu and other respiratory viruses', may be long-lasting.

Moreover, the wearing of masks in public spaces potentially blocks airborne transmission routes. These measures are not being followed uniformly – while overall reductions in transmission may be seen, there may be pockets of decreased or increased transmission, for a variety of reasons, where both COVID-19 and ARO carriage rates are higher.

Within a household setting, prolonged time at home due to COVID-19 interventions could result in increased transmission of other pathogens between household members (Maillard et al., 2020) (e.g. of MRSA [Mollema et al., 2010], *Streptococcus pneumoniae* [Althouse et al., 2017], and MDR-TB [Grandjean et al., 2015; Leung et al., 2013; Paul and Kollikkara, 2014]), resulting in higher carriage levels and more frequent infections with ARO. Alternatively, MDR-TB, a disease also caused by a respiratory pathogen, usually only has 10% of transmission occurring within the home and is likely saturating (Glynn et al., 2015; McCreesh and White, 2018; Middelkoop et al., 2015; Verver et al., 2004; Wilkinson et al., 1997). Hence, an increase in interventions to prevent COVID-19 transmission, and therefore contact outside of the home, could significantly reduce *Mycobacterium tuberculosis* transmission and subsequent MDR-TB incidence.

It is likely that restrictions in normal travel patterns due to COVID-19 will lead to decreased prevalence of carriage with ARO (Murray, 2020). For example, it is known that following travel to countries with high AMR prevalence, travellers can return colonised with bacteria carrying AMR genes and remain colonised for substantial periods of time (Arcilla et al., 2017; ÖstholmBalkhed et al., 2018). Such colonisation likely contributes to the observed international spread of bacteria carrying resistance genes such as those encoding NDM-1 and ‘pandemic’ CTX-M15 conferring resistance to broad-spectrum beta-lactam drugs (Cantón and Coque, 2006; Nordmann et al., 2011; Yair and Gophna, 2018). Change in travel intensity will thus result in reduced introduction and subsequent spread of ARO. However, due to the stochastic nature of such travel-related introductions, it remains unclear whether the success rate of such introductions has or will be affected.

### Health system changes

The COVID-19 pandemic has brought about many changes in both the organisational structures that deliver healthcare services and the way that patients seek care. These are likely to affect antimicrobial usage, altering the populations that are exposed, and hence future prevalence of AMR.

#### Clinical care disruptions

Approximately three-quarters of programmes and service deliveries for HIV, TB, and malaria have been disrupted as a result of the COVID-19 pandemic (Fund, 2020). Similar levels of impact are likely being seen across health systems affecting both infectious and non-communicable disease care such as sexual and reproductive health (Riley et al., 2020). This is motivated by a desire to minimise patient contact due to the potential risk of transmission in overburdened healthcare settings, as well as a repurposing of staff and diagnostic facilities for COVID-19 (Venkatesan, 2020).

This will lead to a disruption in diagnosis – with an increase in time to diagnosis and effective appropriate treatment, those individuals with AROs may be infectious, and hence transmit to others, for longer. For example, South Africa and China have experienced large reductions in TB and MDR-TB testing in the first period of 2020 (Liu et al., 2020; National Institute for Communicable Diseases, Centre for Tuberculosis, 2020). Similarly, with reduced diagnostic capabilities, it could take longer to determine if outbreaks are due to resistant variants, allowing potentially prolonged transmission of, for example, resistant strains of *Salmonella Typhi* (Yousafzai et al., 2019).

For diseases that require long treatment (months, years, or lifelong), such as TB and HIV, clinical care disruptions could lead to interruptions to treatment or provision of longer durations of therapy with less face-to-face support (WHO, 2020k), likely resulting in knock-on antibiotic availability issues (when patients are sent home with more drugs than usual to cover longer periods of time) and suboptimal adherence contributing to relapse. Successful treatment rates for patients with MDR-TB remain low at 56% (WHO, 2020g), hence it is vitally important to prevent disruptions in TB care due to COVID-19 (Stop TB Partnership, 2020). In addition to this, increased stigma for those with diseases mistakenly assumed to be COVID-19 (e.g. TB; Adepoju, 2020) could lead to lower levels of treatment completion, with similar consequences.

Clinical care disruption may lead to a total reduction in in-patient antibiotic prescribing despite high levels of use in those with COVID-19 (Clancy et al., 2020). Like healthcare settings changes that lead to reductions in nosocomial transmission of ARO, the reduction in elective surgeries and healthcare seeking could lead to an overall reduction in antimicrobial use and hence reduction in selection for ARO. Signs of a reduction in use have been seen in healthcare settings in the US, but a concomitantly larger reduction in bed days has sometimes led to an increased number of days of therapy per occupied bed day (Buehrle et al., 2020; Dieringer et al., 2020). Although worrying, this concentration of antibiotic usage in a smaller number of patients may overall exert a lower selective pressure for emergent AMR than wider lower use if these settings reflect patterns seen in wider population use (Olesen et al., 2018).

The widely reported prescription of antibiotics to patients with COVID-19 clashes with the key public health message that antibiotics do not work for viral infections. This could lead to patients in the community having greater expectations of receiving antibiotics for flu-like symptoms, as was the case following the 2009 influenza pandemic (McNulty et al., 2012). With less face-to-face care, and increased telemedicine in HIC, there may be some use of antibiotics in primary care ‘rescue packs’ (Health and Social Care Board (HCSB), 2020) and, where fewer microbiological samples can be taken, more antibiotics prescribed ‘just in case’. A potential signal of this has been seen in data from the UK (Armitage and Nellums, 2020; MacKenna, 2020) and likely increased prescribing for dental care (Croser, 2020). Messages around antimicrobial stewardship need to be tailored to these varied and changing landscapes (De Waele et al., 2021; Seaton, 2020).

The ways in which antibiotics have come to stand in for inadequate healthcare and hygiene infrastructures are becoming better known (Denyer Willis and Chandler, 2019). The prescription or sale of antibiotics in many settings has come to define care when skills, laboratory capacity, financial resources, and time are lacking. Antibiotic use at home has also been documented in response to challenges of daily life (Whyte et al., 2002), with everyday use of, for example, metronidazole operating to curb the diarrhoeal effects of neglected sanitation (Nabirye et al., 2020), and frequent use of multiple antibiotics to counter the symptoms of sexually transmitted infections among sex workers (Nichter, 2001). In a crisis scenario such as the one created by COVID-19, when health services are more stretched and less accessible, the ‘quick fix’ of antibiotics can be expected to increase in both clinical decisions and at home, as has been demonstrated previously, for example, in conflict scenarios (ReAct, 2019). Provided that existing supply chains remain functional, increases in the sale of antibiotics as a workaround for multiple deficiencies in disrupted health systems can be expected.

#### Changes to health-seeking behaviour

In both hospital and community settings, COVID-19 disruptions have led to changes in health- and treatment-seeking behaviour, and potentially difficulties in physically accessing care.

In HICs, where prescriptions are typically required to access antibiotics, this could lead to a short-term reduction in overall antibiotic usage, which may reduce AMR emergence in the short term as well: in the US, estimated total prescription fills for some commonly used antibiotics have been reported to be lower than in 2019 (Vaduganathan et al., 2020). However, in the longer term, those who delay treatment could have worse outcomes, leading to higher rates of hospitalisation and the need for more or different antibiotics. For example, in England there has been an up to 50% reduction in Accident and Emergency attendance in some regions (NHS England, 2020) and a 20% reduction in appointments (Armitage and Nellums, 2020). In the community in England, a reduction in prescriptions for some antibiotics has been seen, for example, for those used to treat skin and urinary tract infections (flucloxacillin and nitrofurantoin and trimethoprim) (MacKenna, 2020), but due to the decrease in the total number of appointments the number of antibiotic prescriptions was higher than expected (Armitage and Nellums, 2020). A substantial proportion of these prescriptions may have been unnecessary, inappropriate antibiotic prescriptions (Pouwels et al., 2018; Smieszek et al., 2018) for self-limiting or viral infections, potentially driven by telemedicine, but again it cannot yet be estimated how many of these patients needed antibiotics and may suffer from later complications. Going forward, local surveillance of the impacts of this heterogeneity in prescribing will be needed to understand both general properties of AMR emergence but also the long-term consequences of this pandemic.

In settings where access to health care is limited, changes in use or delays to seeking health care will have more immediate, potentially drastic consequences. For example, in settings with high levels of sepsis post childbirth, any delay or reduction in utilisation could dramatically increase maternal and child mortality (Kc et al., 2020; Roberton et al., 2020). As discussed above, this could lead to more unregulated antibiotic usage and hence greater selection pressure for AMR, for example, more complex sepsis cases in the future.

To counter this reduction in health-seeking behaviour, some healthcare settings are turning to telemedicine (Croser, 2020; Hollander and Carr, 2020; Rawson et al., 2020b), but the extent to which the inability to see a patient in person leads to ‘just in case’ antibiotic prescribing, and further increases in AMR, is unknown (Armitage and Nellums, 2020). Telemedicine is also not an option in many settings in the world.

#### Vaccination

The fear of attending clinical settings, alongside the disruption to routine immunisation activities due to COVID-19-related burden, has led to reductions in overall vaccination coverage globally (Bramer et al., 2020; McDonald et al., 2020; Saxena et al., 2020; WHO, 2020j).

Vaccines are a key tool to prevent infections, and hence antibiotic use and associated resistance (Lipsitch and Siber, 2016; Malarski et al., 2019; ReAct, 2020; Sosler, 2020). The indirect impact of COVID-19 will be a surge in vaccine-preventable diseases (such as measles; Roberts, 2020) and their associated complications, leading to increased antibiotic use and risk of resistance (GAVI, 2020a). Some vaccines also specifically target resistant subpopulations: for example, serotypes of *S. pneumoniae* with higher resistance to penicillin (Berger et al., 2019; Ginsburg and Klugman, 2017; Klugman and Black, 2018). As with many of the factors here, the impact will be different in different settings. It is likely that vaccine coverage will have decreased more substantially in settings with poorer health systems, where many of these diseases are already at higher prevalence, leading to the potential for outbreaks, increased subsequent antibiotic use, and hence potentially more AMR in these low-income settings, dependent on antibiotic access.

Vaccines are also susceptible to supply issues: the world’s cheapest and safest vaccine, Bacillus Calmette–Guérin (BCG), had global availability and procurement issues from 2013 to 2019 (UNICEF, 2019). BCG immunisation levels have already declined due to COVID-19 health system disruptions in India and Pakistan (Chandir, 2020; Rukmini, 2020). Combined with any supply issues, this could have devastating consequences for TB meningitis levels in children (du Preez et al., 2019). These fragile supply chains may be further tested if current trials exploring the effect of BCG vaccination on COVID-19 provide evidence for efficacy (Curtis et al., 2020).

A positive consequence of COVID-19 has been an emphasis on avoiding a dual ‘flu and COVID-19' health burden, leading to increased coverage of influenza vaccination (e.g. in England; Public Health England, 2020c). This will have the knock-on effect of reducing antimicrobial prescribing, both inappropriate and appropriate, for influenza-like infections and secondary bacterial complications (Klein et al., 2020; Knight et al., 2018a).

As with the influenza vaccine above, the new COVID-19 vaccines (Parker et al., 2020) have the potential to remove much of the antibiotic use currently used directly for those with COVID-19. However, vaccination programmes are slow and highly variable: compared to rapid programmes already started in HICs, many low-income countries will have few vaccines doses in 2021 (Dyer, 2020). Issues such as vaccine hesitancy are also a concern (de Figueiredo et al., 2020; Lazarus et al., 2020; Schmid et al., 2017). Vaccine hesitancy is context-specific, and while previous pandemic vaccination campaigns have met with high rates of vaccine acceptance, for example, for cholera (Lucas et al., 2005) or some measles outbreaks (Grais et al., 2008), this may not be the case for COVID-19. Combined, these issues may mean that coverage of COVID-19 vaccination is lower in certain subpopulations or geographical settings that already have high antibiotic use and hence high ARO prevalence, or low coverage of existing vaccines, potentially magnifying disease burden. Practically, then, even with reported sensitivities of some COVID-19 vaccines exceeding 90%, there may be local outbreaks and rolling local-level lockdowns that hamper public health efforts, full economic recovery, and hence AMR control for several years.

## The future of AMR research

We have described a range of intersections between AMR and the COVID-19 pandemic. Our global public health agenda will have to support the management of problems related to COVID-19 in conjunction with existing challenges such as AMR for the foreseeable future. The WHO has recently published measures to ensure that antimicrobial stewardship is integrated into pandemic responses (Getahun et al., 2020).

The range and scale of changes described in this article hold the potential for a step change not only in the emergence of AMR but also in transmission and ultimately in the burden for different populations (Figure 1). In response, we outline below a series of research priorities that address the need to anticipate and ameliorate the effects of the COVID-19 pandemic for AMR. In considering research needs, we begin by drawing attention to the impacts of COVID-19 on AMR research activity and the potential for mitigation.

### Impact of COVID-19 on AMR research

AMR research is likely to have slowed substantially in the first half of 2020. Staff, equipment such as PCR machines for diagnostic laboratories (Cepheid, 2020; Department of Health and Social Care, 2020), and reagents have been redeployed to COVID-19 research. Physical distancing and logistical issues (shielding concerns, travel restrictions, or childcare) will severely limit the amount of laboratory science that can be performed. Indeed, the switch to working on COVID-19 has affected all disciplines involved in AMR research. The impact of this will not only be interruption to crucial research that would otherwise have been performed, such as clinical trials that have been put on hold (Cleary, 2020; Rusen, 2020; Thornton, 2020), but gaps in our monitoring of AMR: with the pandemic pressure of COVID-19, many routine microbiology samples that are used for global AMR monitoring such as Global Antimicrobial Resistance Surveillance System (GLASS; WHO, 2020c) will be missing for 2020. This rapid mobilisation of research power may leave AMR research unsupported without top-level prioritisation and support (Hsu, 2020).

#### Mitigation strategies for AMR research

To combat this, researchers should be supported to continue AMR work. We must also build on the push that the COVID-19 pandemic has given to making science even more open, with results shared across scientific groups and institutes (Gold, 2020). The increasing use of preprint servers and sharing of early results should be built into future research plans, alongside the further development of strategies, to ensure that conclusions based on these outputs are reviewed following critical assessment through the peer-review process. The impact preprints have had on the science of COVID-19 suggest that in the future greater caution should be exercised when the study results may directly affect treatment of patients (e.g. reports on drugs or vaccines) (Flanagin et al., 2020).

New networks of collaborators formed to tackle COVID-19 issues (such as in One Health and hygiene [CGIAR, 2020; COVID-19 Hygiene Hub, 2020]) should also be utilised in future research plans to tackle similar interdisciplinary problems in AMR. COVID-19 has demonstrated once again the importance of multidisciplinary teams, showing how disciplines can successfully collaborate – they provide valuable complementary insights that should be harnessed for AMR.

COVID-19 has also highlighted how rapid the development and assessment of treatments and vaccines can be, with novel platforms and adaptive designs that should now be applied to antibiotic development. In particular, care must be taken to build on this for all antimicrobials and to make sure that a focus on supporting antivirals or vaccine development at the Pharmaceutical, Government and Global organisation level (GAVI, 2020b) does not result in fledgling antimicrobial development pipelines failing. Companies developing novel antimicrobials were already susceptible to failure (Mancini, 2020), and investors may have even lower enthusiasm for antimicrobial development during and after the COVID-19 pandemic, compounded by the economic crisis. Governments are intervening in this area; the US has developed legislation in the area of antibiotic funding mechanisms (The Pioneering Antimicrobial Subscriptions to End Upsurging Resistance Act) (Bennet and Young, 2020; Clancy and Nguyen, 2020). In the UK, a new scheme to pay pharmaceutical companies upfront for their work on antimicrobial development has been developed (UK Government, 2020), with the first two new antibiotics having been chosen in December 2020 (Perkins and Glover, 2020). While there are substantial limitations to these approaches (Glover et al., 2019), they demonstrate that governments understand the importance of AMR and are working to mitigate the market failures in this area.

Going forward, there is a greater appreciation of the consequences of the spread of an untreatable ARO: SARS-CoV-2 highlights how difficult such a pathogen would be to control once it has emerged (Murray, 2020). In turn, this greater awareness is likely to lead to more support of research into anti-infectives (Engel, 2020; McKenna, 2020).

### Research and health system priorities for AMR

#### Diagnostics

COVID-19 has highlighted both the possibilities and limitations of diagnostic technologies; while in many settings diagnostic logistics with built-in redundancies have been rapidly scaled up, globally we are struggling to diagnose SARS-CoV-2 infection reliably and rapidly.

Moving forward, the AMR community should capitalise on the investment in diagnostic logistics and adapt their use for rapid detection of both the causative agent of infection and any associated drug resistance, especially in LMICs. For example, across Ghana, capacity building (both laboratory and personnel) is needed to tackle both COVID-19 surveillance and guide antimicrobial prescribing (Egyir et al., 2020). Similarly, the diagnostic uncertainties faced in COVID-19 are common concerns for bloodstream infections, suspected pneumonia, and other areas where healthcare workers are required to differentiate between bacterial and viral infections. The AMR community should support the current momentum to develop and make widely available a cheap, reliable, and rapid point-of-care test (such as rt-LAMP; Thompson and Lei, 2020) to detect viral infections such as the ACT-Accelerator Diagnostics Partnership. Differentiating bacterial from viral infection and rapidly determining resistance carriage would not only reduce unnecessary antibiotic prescribing and hence AMR emergence (Getahun et al., 2020; Patel et al., 2020), but also reduce onward transmission of ARO. Importantly, research should be into not only the technology but into the implementation of diagnostics, and appropriate diagnostic stewardship, since lessons learned from the COVID-19 diagnostic scale-up have shown that more diagnostics are not always the solution they are hoped to provide (Patterson, 2020).

#### Monitoring

COVID-19 has once again highlighted that data collection for infectious diseases needs to be strengthened, open access, and active. This is particularly important for AMR which often relies on passive surveillance: WHO’s GLASS is moving to active monitoring but currently takes convenience samples of those isolates that have been sent for phenotypic resistance testing. This means that they are biased towards sampling individuals with complex, persisting infections that are more likely to be caused by resistant pathogens. Global AMR monitoring is further hampered by large gaps: notably low-income regions such as sub-Saharan Africa lack national AMR surveillance capacity and quality assurance of laboratory procedures (Seale et al., 2017; WHO, 2018).

New monitoring systems developed and implemented for COVID-19 could potentially be harnessed to reinforce environmental monitoring of AMR. For example, surveillance of waste water could be used for the early detection of both changes in COVID-19 and ARO prevalence (Bivins et al., 2020; Hendriksen et al., 2019), as well as antibiotic concentrations (Comber et al., 2020).

The costs for screening for COVID-19 have, in most settings, been born by governments. However, screening for drug-resistant pathogens, such as CRE, at hospital admission, is usually paid for by hospitals or patients themselves. Understanding the funding model and how it could change, potentially with the argument of the wider economic impact of ARO prevalence, could lead to better screening and hence monitoring of ARO prevalence.

The heterogeneity in COVID-19 outbreaks at the sub-national level has furthermore emphasised the need for fine scale local monitoring of the public health situation. To understand the impact of COVID-19 on AMR, we will need fine measurements of antimicrobial use, rapidly shared, not just within primary and secondary care for example, but between different patient groups within hospitals. We do not currently have this granularity and hence, nearly a year into this pandemic, do not know either how antimicrobial use has changed or how AMR prevalence has been altered. This is even though diagnostic labs may have been some of the only services that have been functioning in recent months, although often overwhelmed with COVID-19 demands. Without data, countries cannot rapidly tackle issues at the local level needed to control spread since local capacity is needed to rapidly respond to both COVID-19 and AMR on the ground.

The many interactions we have described here will have interlinking effects but will also act on different timescales. Short-term shortages of certain antimicrobials will only have an impact on levels of resistance to that antimicrobial if resistant strains are somehow less ‘fit’ than their susceptible counterparts (Björkman and Andersson, 2000). A reduction in elective procedures may prevent transmission of hospital-associated ARO leading to potential elimination from a setting or may only reduce levels in the short term. Changes in antibiotic use at varying timescales across settings could potentially allow for the unpicking of the relative contributions of settings to overall AMR prevalence (e.g. community vs. hospital settings; Knight et al., 2018b). The impact in high-income settings of the opposing effects of prolonged reduced community transmission versus high but short antibiotic use in a small subset of populations (e.g. hospitalised COVID-19 patients) suggests optimism for future AMR emergence against a background of potentially higher morbidity or mortality due to cancelled elective procedures. However, the longer-term undermining of health structures, for example, could lead to increased drug pressure and transmission of AMR. Detailed monitoring and analysis of such dynamics will provide not only information on treatment options, but also insight into the dynamics of resistance evolution.

#### Antimicrobial usage

Much of the high antimicrobial use in patients with COVID-19 is unavoidable (as discussed above). Research is now needed into better understanding risk factors and prevalence of co-infection in patients with COVID-19 to support a reduction in any avoidable antimicrobial prescribing. Equally, with the collection of better, more granular data, we can improve empiric antimicrobial stewardship guidance based on local ARO prevalence. This will lead both to reductions in AMR emergence but also improved patient outcomes.

Aligned with the above, we need good data on what antimicrobials are being used or prescribed for what, where, and when alongside environmental data on residual concentrations. For example, the impact of shortages on the use of protected categories of antibiotics, such as the Watch and Reserve in the AWaRE classification, remains unclear (WHO, 2019). Moreover, data are needed on the use of such therapeutics claimed to work for COVID-19 but shown to no longer be efficacious (such as hydroxychloroquine and azithromycin) across all settings.

As a community, we need to ensure that the potential individual benefits of antimicrobial prescribing are weighed against the population impact of AMR emergence. Existing efforts to optimise empiric prescribing and support adherence to well-designed locally specific guidelines need to be tailored to emergency situations (Stevens et al., 2020). In such scenarios as presented by COVID-19, the trade-offs between presumptive and targeted prescribing shift. The risks of not prescribing can be increased within fragmented systems with reduced access to care and therefore projections of the amount of increased presumptive antibiotic use that could be tolerated are required, together with analysis of which antibiotics may be more and less tolerable to increase in use for the longer-term AMR picture. Such analyses need to recognise both a short-term scenario of COVID-19 impacts as well as the longer-term recession that may impact some geographies.

Research is also needed to clarify the role of biocides in selection of AMR so that they can be used appropriately in both healthcare and community settings (Getahun et al., 2020; Maillard et al., 2020).

#### Health system strengthening

COVID-19 has highlighted many weaknesses in our health systems. The WHO has called for a broader pandemic response that 'ensures the continuity of essential health services and regular supply of quality assured and affordable antimicrobials including antiretroviral and TB drugs, and vaccines’ (Getahun et al., 2020). Health systems research into specific regulatory and contracting mechanisms to prevent stockouts and supply chain disruptions of generic and non-generic drugs, as well as vaccines, is urgently needed. Similarly, the network logistics under development to deliver COVID-19 vaccines need to be designed to bolster other vaccine campaigns in order to protect vaccine effects on AMR. COVID-19 has highlighted the need for better health service infrastructure with greater resilience to deal with shocks such as outbreaks. How best to create these with limited resources requires further research.

More broadly, urban informal settlements are potential AMR ‘hotspots’ due to high population density and lack of water supply and sanitation infrastructure (Nadimpalli et al., 2020), which could also lead to enhanced COVID-19 transmission and severe impacts among already vulnerable populations (Corburn et al., 2020). Cost-effectiveness analyses are required to support prioritisation for investment in infrastructure and public health prevention strategies, taking potential synergies into account to address these two public health threats.

#### Prioritisation

As we move forward, one way to mitigate the impact of COVID-19 on AMR is to ensure that priorities for public health are established in line with key measures such as mortality burden.

One impact of this pandemic will be to shake up public health agendas in HICs where non-communicable diseases have been dominating: the COVID-19 pandemic has acted as a reminder to us all of the human vulnerability to emerging infectious diseases (Fauci et al., 2020). This reminder was not needed in some low-resource, vulnerable contexts, where recurrent outbreaks of infectious diseases, such as the Ebola virus disease in the Democratic Republic of the Congo, keep infectious diseases high on the list of preoccupations and public health priorities. As such, existing response structures and mechanisms may be well placed to help control the COVID-19 pandemic (Dzinamarira et al., 2020; Mobula et al., 2020; Nachega et al., 2020) or indeed any future ARO emergence (Rawson et al., 2020a). The thematic overlaps between COVID-19 and an ARO should be emphasised as the AMR community moves forward, rather than competition for scant resources.

In considering the interplay of these two public health priorities, it is important to consider the relative burdens by country. In some settings, COVID-19 may not be the top priority in terms of infection or disease burden. For example, in Viet Nam, the number of COVID-19 cases is currently low (1539 cases as of 20 January 2021; WHO, 2020h), but in 2019 there were 3234 confirmed cases of MDR-TB alone (WHO, 2020f). In Greece, the number of COVID-19 deaths is also relatively low (5488 deaths as of 20 January 2021; WHO, 2020h), and hence a more pressing public health concern could be that approximately 40% of all infections in 2018 were estimated to be due to antibiotic-resistant bacteria (OECD, & ECDC, 2019). In particular, the Greek incidence of infections with carbapenemase-producing Enterobacteriaceae is the highest of all European Union (EU) and European Economic Area (EEA) countries (Cassini et al., 2019; Grundmann et al., 2017). In other settings with comparatively low infectious disease or wider AMR burden but a large outbreak of COVID-19, such as the UK and the﻿US, COVID-19 is the current acute public health priority. Of most concern are those countries, such as India, with a high burden of both AMR and COVID-19. With more than 10.5 million confirmed cases of COVID-19 in January 2021 (WHO, 2020h), and one of the largest number of AMR infections globally (Gandra et al., 2017; Gandra et al., 2019; Laxminarayan and Chaudhury, 2016), the potential for mutual exacerbation is significant.

The issue going forward is that COVID-19 continues to be a major disruption to not only public health but to the way our societies function in general. We cannot use disease burden in a country to directly translate to public health priority setting – those with low COVID-19 case numbers have, in part, been those that have treated it as a priority. However, the future public health agenda requires refocus, with a balancing act of countering COVID-19, and COVID-19 collateral, against prior public priorities such as AMR.

#### Inequality

Across the world, inequalities are rising between and within countries (Institute for Policy Studies, 2020). COVID-19 has rendered the health impacts of these inequalities visible and palpable in tragic terms. Research now needs to take forward these recognitions of the numerous lines through which health inequalities operate when understanding both infectious and other causes of disease. The layering of multiple dimensions of marginalisation at the intersections of race, gender, and class needs to inform research and intervention strategies that recognise the compounding effects for AMR and COVID-19 among other conditions that emerge as much through lack of access to equal care and opportunity as they do from living conditions that privilege some above others in health outcomes (Glover et al., 2020). The concept of syndemics can usefully be deployed to conceptualise these compounding and interacting effects of the health, social, economic, and political worlds (Mendenhall, 2020).

As a scientific and public health community, we need to ensure that as we intervene to prevent COVID-19 we are not exacerbating pre-existing inequities, nor creating new ones (Glover et al., 2020). Research must be designed to capture these potential effects, and, critically, such research must be linked closely to policy in order to enable change in direction of initiatives in the light of new evidence. Developing these approaches also presents the opportunity to expand learning to broader infectious disease interventions that have as yet failed to address the uneven distribution of disease burden including with AROs. In particular, we must determine if any existing inequalities lead to synergistic, multiplicative impacts on both AMR and COVID-19, and work to develop and evaluate mitigation strategies at all levels.

#### Policy research

The pandemic has exposed weaknesses in our national and supranational organisations such as the US Centers for Disease Control and Prevention (CDC), Public Health England, and the WHO, for example, in their funding model. These public health bodies are key to coordinating AMR responses through data gathering, standardisation, and policy guidance. It would be disastrous for the AMR response should countries decide to go their own way in these areas. As such, work needs to be done to support these organisations – we especially need to maintain confidence in the WHO to enable national AMR programmes to operate. This confidence is also crucial in the fight against misinformation: we need global agencies to be the accepted ‘gold standard’ public health evidence base, communicating the evidence widely and clearly to all members of society (Arshad et al., 2020).

Worryingly, a recent report from the WHO Health Emergencies Programme found no clear evidence that the formation of National Action Plans (NAPs) for Health Security relates to a country’s ability to respond to COVID-19 (Independent Oversight and Advisory Committee (IOAC), 2020). There are huge implications if the same holds true for AMR, where NAPs are viewed as the cornerstone of our ability to monitor and control the problem. Lessons should be learned from the NAPs for Health Security and a clear strategy designed to test NAPs for AMR building on the current monitoring of progress towards implementation of the global plan (WHO, 2020a).

The importance of such global bodies in coordinating the international ability to tackle global health crises is highlighted by the success of the WHO COVID-19 Solidarity Response Fund as well as the COVID-19 vaccine fund (GAVI, 2020b; WHO, 2020b). The AMR community needs to work to prevent such funds ever being needed for an ARO – primarily by the research outlined above, but also by designing and supporting policy guidance and implementation research for AMR.

## Conclusion

This review demonstrates that, through modifying the processes of emergence, transmission, and infection burden, COVID-19 is changing the landscape of AMR. A simplified view might be that AMR will be reduced in the face of the efforts made to curb COVID-19 or that the use of antimicrobials to treat COVID-19 will increase ARO prevalence. However, we have outlined that the impacts on AMR of the varied responses to the SARS-CoV-2 virus in different settings are difficult to predict and will vary in the short, medium, and long term. As we move forward, vaccination and rapid tests may reduce the burden of COVID-19 and hence the initial dramatic impact on antibiotic use, but the scars in our health systems may take a long time to heal, leading to altered antimicrobial use in many settings going forward. We make the case for finely tuned research that measures and responds to the crisis of COVID-19, with future systems that support continued antimicrobial stewardship.

Though the public health community has been focused on the global COVID-19 pandemic, AMR has not gone away. In fact, many of the issues we face during the pandemic are related to the AMR landscape. As health systems struggle to cope with this new disease, the old ones and their drug-resistant subpopulations need to be kept in public health focus. The AMR community needs to take care to see the impact of COVID-19 on AMR from the global perspective – the challenges and resulting impact on AMR will be different by country as well as by setting (Clancy et al., 2020; Collignon and Beggs, 2020; Monnet and Harbarth, 2020; Pokharel et al., 2019).

While the impact of COVID-19 policies on drug-resistant pathogens is still unknown, what is clear is that there will be a shifted set of AMR global challenges going forward. We now need to work together as a multidisciplinary community to gather data on these changes and solve the arising challenges collaboratively.
